# Depth‐wise multiparametric assessment of articular cartilage layers with single‐sided NMR

**DOI:** 10.1002/nbm.5287

**Published:** 2024-11-07

**Authors:** Carlo Golini, Marco Barbieri, Anastasiia Nagmutdinova, Villiam Bortolotti, Claudia Testa, Leonardo Brizi

**Affiliations:** ^1^ Department of Physics and Astronomy University of Bologna Bologna Italy; ^2^ Department of Radiology Stanford University Stanford CA USA; ^3^ Department of Civil, Chemical, Environmental and Material Engineering University of Bologna Bologna Italy

## Abstract

Articular cartilage (AC) is a specialized connective tissue that covers the ends of long bones and facilitates the load‐bearing of joints. It consists of chondrocytes distributed throughout an extracellular matrix and organized into three zones: superficial, middle, and deep. Nuclear magnetic resonance (NMR) techniques can be used to characterize this layered structure. In this study, devoted to a better understanding of the NMR response of this complex tissue, 20 specimens excised from femoral and tibial cartilage of bovine samples were analyzed by the low‐field single‐sided NMR‐MOUSE‐PM10. A multiparametric depth‐wise analysis was performed to characterize the laminar structure of AC and investigate the origin of the NMR dependence on depth. The depth dependence of the single parameters *T*_1_, *T*_2_, and *D* has been described in literature, but their simultaneous measurement has not been fully exploited yet, as well as the extent of their variability. A novel parameter, *α*, evaluated by applying a double‐quantum‐like sequence, has been measured. The significant decrease in *T*_1_, *T*_2_, and *D* from the middle to the deep zone is consistent with depth‐dependent composition and structure changes of the complex matrix of fibers confining and interacting with water. The *α* parameter appears to be a robust marker of the layered structure with a well‐reproducible monotonic trend across the zones. The discrimination of cartilage zones was reinforced by a multivariate principal component analysis statistical analysis. The large number of samples allowed for the identification of the smallest number of parameters or their combination able to classify samples. The first two components clustered the data according to the different zones, highlighting the sensitivity of the NMR parameters to the structural and compositional variations of AC. Using two parameters, the best result was obtained by considering *T*_1_ and *α*. Single‐sided NMR devices, portable and low‐cost, provide information on NMR parameters related to tissue composition and structure.

## Graphical abstract

Single‐sided nuclear magnetic resonance assessment of articular cartilage structural changes along layers is possible. The measured trends of *T*_2_, *T*_1_, *D*, and *α* parameters highlight their sensitivity to the organization and content of chondrocytes, collagen, and proteoglycans that lead to different conditions of restrictions to water molecule diffusion.

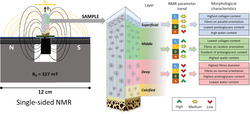

**Abbreviations**
AC: articular cartilage
DQ: double‐quantum
ECM: extracellular matrix
FDR: false discovery rate
KW: Kruskal–Wallis
MOUSE: Mobile Universal Surface Explorer
NS: Not Significant
OA: osteoarthritis
PBS: Phosphate Buffered Saline
PCA: principal component analysis
PG: proteoglycan
PTFE: polytetrafluoroethylene
RF: Radiofrequency
SNR: signal‐to‐noise ratio
UPEN: Uniform‐Penalty

## 1 INTRODUCTION

Articular cartilage (AC) is a highly specialized connective tissue that covers the ends of long bones within the synovial joint cavity. The tissue facilitates the load‐bearing of joints by cushioning the underlying subchondral bone from excess stresses and distributing the load during joint movements. The mechanical features of AC provide minimal friction and excellent lubrication within the joint. These physical and mechanical properties are based on the specific chemical composition and metabolic activity of the tissue, which consists of a relatively small number of highly specialized cells, the chondrocytes, distributed throughout an abundant extracellular matrix (ECM) substance responsible for communication between cells.^1^, ^2^, ^3^

The composition, organization, and mechanical properties of the ECM, cell morphology, and cell function vary according to the depth with respect to the articular surface. The morphological changes in chondrocytes and matrix from the surface to the calcified and subchondral zones make it possible to define three regions^2^: superficial, middle (or transitional), deep (or radial), and then the calcified region. The relative size and appearance of these zones vary among species and joints within the same species: although each zone has different morphological features, the boundaries between them cannot be sharply defined. Nonetheless, zones differ concerning concentrations of water, proteoglycan (PG), and collagen and concerning the size of the aggregates. Cells in different zones differ in shape, size, and orientation relative to the articular surface and in metabolic activity^3^ as well. In human cartilage, collagen and PG contents increase as a function of tissue depth.^4^ Such inhomogeneity produces depth‐wise differences in the response of the tissue to tension and compression.

A common cartilage disease causing the loss of AC is osteoarthritis (OA). In the setting of OA, AC undergoes compositional degradation that leads to a change in its mechanical and metabolic properties. In early‐stage OA, cartilage degeneration is primarily characterized by PG loss and collagen deterioration, which impairs normal mechanical and metabolic properties of the tissue and leads to cartilage loss with disease progression. Furthermore, there is experimental evidence that cartilage degeneration is depth‐dependent,^5^, ^6^, ^7^, ^8^, ^9^, ^10^ and accounting for such depth‐wise changes improves biomechanical modeling of knee OA progression.^11^ Thus, obtaining compositional depth‐wise information on cartilage may be crucial for advancing our understanding of this common and degenerative disease, especially if paired with biomechanical testing and modeling.

Radiography is the most deployed imaging modality for studying the integrity of the cartilage tissue and its modifications, and represents the gold standard for establishing an imaging‐based diagnosis of OA.^12^ However, radiography can only assess changes in cartilage morphology, which are secondary to compositional changes, limiting its value in detecting early‐stage of the disease. Furthermore, other limitations of radiography are the lack of sensitivity and specificity for the detection of articular tissue damage.^13^ An alternative technique that could be used is nuclear magnetic resonance (NMR). NMR provides many promising techniques (MRI [magnetic resonance imaging], relaxometry, diffusometry, field cycling), applied both singularly and in combination, for noninvasive characterization and evaluation of AC and for diagnosing and monitoring of the degenerative progression.^14^

In the present study, for the sake of simplicity, we will use the terms *T*_1_, *T*_2_, and *D* for the longitudinal and transverse NMR relaxation time and self‐diffusion coefficient, respectively, instead of the more appropriate terms *observed* or *effective* or *apparent T*_1_, *T*_2_, and *D*.

In vivo MRI studies have shown the possibility of evaluating cartilage composition by the relaxation times (i.e., *T*_1_, *T*_2_, *T*_2_*, and *T*_1ρ_)^15^, ^16^ exploiting contrast agents, sodium imaging, and magnetization transfer techniques.^17^

However, in vivo quantitative MRI usually provides limited resolution within the cartilage structure of a few millimeters. Despite recent developments that allow quantification of *T*_2_ ^15^, ^16^ and *D* ^18^ in cartilage with an in‐plane resolution of a few hundred micrometers, it is infeasible from a practical point of view to obtain an evaluation of all the NMR parameters (henceforth, multiparametric information). Therefore, ex vivo studies that allow depth‐wise characterization of cartilage are still an essential tool for studying cartilage composition and changes related to pathologies of AC.

Ex vivo microscopic MRI (μMRI) provides images with a spatial resolution of up to tens of microns of different NMR parameters, including signal intensity, *T*_1_, *T*_2_, and *D*, and is especially useful for heterogeneous systems, such as AC.^19^ An unavoidable drawback is that only small samples (linear size of a few mm) can be analyzed by devices providing such a high resolution. μMRI at the resolution of 14 μm showed three regions in AC, corresponding approximately to the three known zones of cartilage. Moreover, it demonstrated a *T*_2_ anisotropy explaining the laminated appearance in MRI.^19^, ^20^, ^21^ Also, changes induced by loading on topographical distribution of the zonal properties of OA tibial cartilage have been studied by μMR,^22^ also in combination with polarized light microscopy.^23^, ^24^

Time domain NMR relaxometry (TD‐NMR) techniques have long been used to study cartilage ex vivo properties. In TD‐NMR studies of collagen in bone and cartilage,^25^, ^26^, ^27^ the acquired free induction decay appeared to be the sum of an initially approximately Gaussian (*T*_2_ about 20 μs) component and a longer exponential component. The two spin pools correspond to the so‐called low‐ and high‐mobility ^1^H nuclei, simply called “solid” and “liquid,” ascribed to ^1^H nuclei of restricted mobility, likely ^1^H of collagen (covalently‐bound, e.g., CH and CH2), and of bound and free water molecules, respectively.^25^, ^26^, ^27^ In these studies, cartilage was considered as a porous medium saturated by water, where internal surfaces, largely associated to the “solid” ^1^H nuclei, can contribute to longitudinal relaxation of ^1^H of water molecules.^28^ In particular, the description^25^, ^26^, ^27^ was consistent with the observation of the same *T*_1_ for the two pools.

The solid signal with such a short *T*_2_ has been described recently in ref.^29^, where two‐dimensional (*T*_1_‐*T*_2_) measurements of bovine AC samples were carried out at different stages of hydration. The *T*_1_‐*T*_2_ correlation maps showed that the two pools have the same *T*_1_.^29^ The “solid” pools have been studied in AC by magic angle spinning NMR.^4^ In ref.^30^, two macromolecular populations corresponding to collagen and PG in human cartilage have been identified through their diffusive properties. Moreover, by *T*_1_–*T*_2_ correlation studies, two populations were distinguished, corresponding to solid and liquid components. In ref.^31^, human AC samples at stage IV (last stage) of OA were analyzed with spatially resolved pulsed gradient stimulated echo NMR, and it was shown that the diffusion of fluid and biopolymers are sensitive to OA.

Single‐sided NMR^32^, ^33^, ^34^, ^35^, ^36^ devices combine the capabilities of TD‐NMR and NMR diffusometry methodologies with the possibility of obtaining one‐dimensional high‐resolution spatial information (order of 100 μm). Thus, they may provide an appealing approach for assessing the biological properties of tissues.^35^ In fact, single‐sided devices have demonstrated their potential to study both soft and mineralized tissues.^30^, ^32^, ^35^, ^37^, ^38^, ^39^ These devices allow signal detection from a sensitive volume (a slab), suitably selected inside a sample placed on top of the magnet surface. Single‐sided devices have the additional advantages of low acquisition, running, and maintenance costs, as they consist of small permanent magnets and are portable. Many studies on AC have been reported using these devices. *T*_2_ and *T*_1_ relaxation times measured in low fields and with high spatial resolution allow one to distinguish the three cartilage layers.^40^ High‐resolution one‐dimensional (profile) relaxation imaging was obtained on AC,^41^ and *T*_1_ profiles coupled with relaxation dispersion measurements were performed on ex vivo human samples affected by OA.^42^

Combining single‐sided studies with other NMR techniques can give further insights into cartilage structure. A multicomponent *T*_1_ analysis^43^ and variable field studies of molecular dynamics^29^ in AC have also been performed. In ref.^44^, relaxation dispersion data were acquired to study the cross‐relaxation effect of protons with ^14^N nuclei in proteins, and a reduction of the cross‐relaxation contribution observed in OA was considered predominantly due to the increased water concentration in OA. Human knee cartilage samples were studied in ref.^45^ by low‐field single‐sided profile and variable‐field NMR relaxometry with and without applying pressure to the samples. The quadrupolar dips observed in the ^1^H relaxation time dispersions and the thickness changes under load showed the strongest correlation with the Mankin score in OA.^45^

These studies have revealed the capability of single‐sided NMR devices to characterize cartilage structure and demonstrated their potential to be sensitive to possible changes induced in tissue. Because different NMR parameters are sensitive to different elements of cartilage composition and structure, it is possible that a combination of NMR parameters could lead to a better depth‐wise characterization of cartilage. Further, a multiparametric approach could help differentiate tissues coming from different sub‐regions of the joint. Nonetheless, such a multiparametric approach has not been fully exploited yet. Furthermore, to compare preclinical studies, standardization would be needed in sample preparation to minimize the cartilage dehydration occurring during NMR measurements. Differences in protocols may significantly affect experimental results, as changes in the hydration state of cartilage lead to changes in the tissue and, thus, its NMR properties, which can only increase the inevitable divide between in vivo and ex vivo experiments. Despite the lack of standardization, it is known from literature^19^, ^31^, ^40^, ^46^ that the behavior of the NMR profile can identify the three layers in AC: the regions of initial increase of relaxation time (*T*_1_ and, or *T*_2_, as it will be better discussed later), higher values, and subsequent decrease towards the calcified zone correspond to superficial, middle, and deep layers, respectively. It is worth noting that the change in the direction of the magnetic field with respect to the cartilage surface can alter the *T*_2_ value.^19^, ^20^, ^40^, ^47^

The aim of the present study was a depth‐wise characterization by multiple NMR parameters of the layered structure of AC using a commercially available single‐sided NMR device. The study was conducted on samples ex vivo trying to preserve as much as possible cartilage hydration during the timeframe required to perform the entire set of multiparametric NMR experiments. Several experimental protocols were tested to select the optimized experimental set‐up to carry out the central object of the study: to investigate the ability of four NMR parameters, considered singularly and in combination, to characterize the layered structure of cartilage samples cored from different locations of bovine knee joints. Specifically, the NMR parameters investigated were the ^1^H NMR *T*_2_ and *T*_1_, the water molecules *D*, and a novel parameter *α* derived from applying a build‐up double‐quantum‐like (DQ) sequence.^34^, ^48^ Due to the nature of the sequences and device used, the values of the NMR parameters originate from liquid ^1^H, namely, water and PG. A specificity of this study is the rather large number of samples analyzed by a multiparameter approach. The knowledge about the scatter of many concurrent NMR parameters is of utmost relevance for the study of cartilage tissues and for the ultimate goal of the identification of diseases. The large experimental data collection allows for a statistical analysis able to identify the smallest number of parameters or their combination able to classify samples. This is a further novelty of this study. The feasibility of applying the build‐up DQ‐like sequence in the study of cartilage with single‐sided NMR constitutes another novelty of this work.

## 2 EXPERIMENTAL SECTION

### 2.1 Sample extraction and treatment before NMR measurements

Osteochondral specimens were extracted from skeletally mature bovine knees from six animals. Articular segments were obtained from the food supply chain; no animals were sacrificed for the purposes of this work. The coring procedure of the joints was performed by the Laboratory of Medical Technology of the IRCCS Rizzoli Orthopedic Institute (Bologna, Italy). Each knee (frozen immediately after slaughtering) was thawed at about 5°C for 15 h. Four pieces were obtained from each knee: two tibial plateaus and two femoral condyles. Then, 40 cylindrical cores (diameter, ∅ = 10 mm, and height, *h* = 10 mm) were extracted (about the same number for each region). Cartilage thickness within the samples ranged approximately from 1 to 3 mm. These samples were analyzed without removing them from the osteochondral unit, with a view to future mechanical analysis on the whole package of articular tissues. To preserve the moisture of AC, immediately after being cored, the samples were immersed in a phosphate‐buffered saline (PBS) solution (concentration = 1.0×, pH = 7.4) and then stored in a freezer. The samples were then defrosted one at a time in the following days for NMR measurements. Before NMR measurements, the samples were first thawed for 20 min at room temperature inside the PBS solution. Samples were then put inside an NMR glass tube (diameter 10 mm), as described in Section 2.3. To study the changes in NMR parameters following the freeze–thaw procedure, a set of five samples was measured immediately after coring, skipping the freezing procedure. The distributions of most parameters are in agreement with those measured in the freeze‐thawed samples (see Supplementary Material Figure).

Twenty samples were used to perform the NMR multiparametric experiment, the central object of this study, while the remaining samples were preliminarily used to optimize the protocols for both the sample preparation, the NMR data acquisitions, including the radiofrequency (RF) sequences, and the automatic procedure for layer definition and selection.^49^, ^50^

### 2.2 NMR acquisition

**FIGURE 1:**
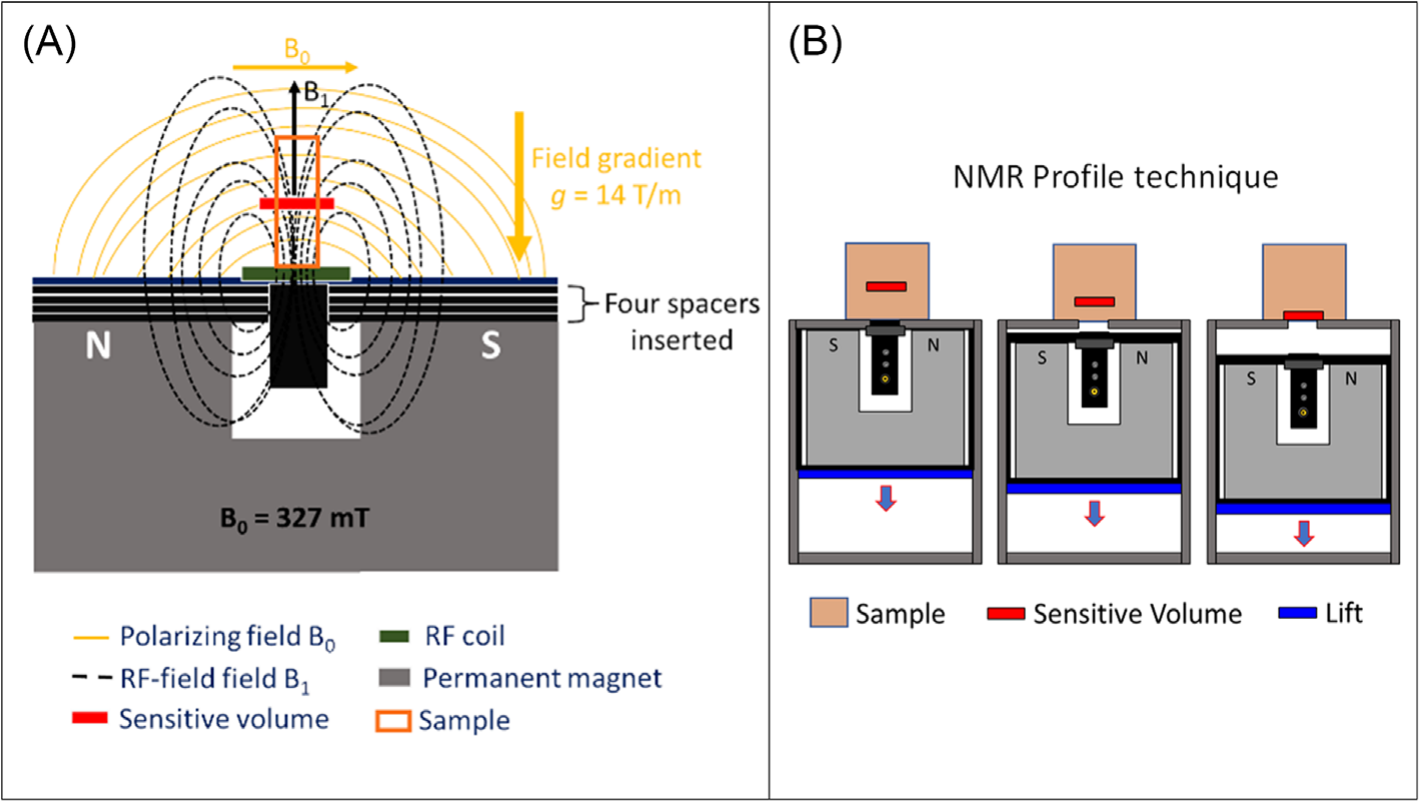
(A) Schematic representation of the probe system that supports the sample. The *B*_1_ RF field, the static *B*_0_ polarization field, and the sensitive volume are depicted. In this work, the considered sensitive volume had a size of (15 × 15 × 0.21) mm^3^. (B) Sketch of the NMR profile technique by the micrometric‐precision lift. NMR, nuclear magnetic resonance; RF, radiofrequency.

The single‐sided Profiler NMR‐MOUSE PM10 (Figure 1) (polarizing magnetic field strength *B*_0_ = 0.327 T, Larmor frequency 13.8 MHz) equipped with the KeaII spectrometer and a high‐precision lift for automatic vertical movement of the permanent magnet (Magritek, NZ) was used for the nondestructive investigation of the specimens. The Kea Prospa software (Magritek, NZ) controlled RF generation and pulse programs. A constant gradient of the polarizing field is present along the direction perpendicular to *B*_0_, with a gradient intensity of 14 T/m, uniform over the sensitive volume (Figure 1). The sensitive volume is the region in which the static gradient causes protons to resonate in a band of frequencies matching the bandwidth of the RF pulses. The lift enabled the acquisition of the so‐called NMR profiles and to perform the measurements of the four parameters along the depth of the cartilage sample. This analysis determines the different zones to be identified with the anatomical zones. Four 2 mm spacers were inserted between the permanent magnet and the surface coil (Figure 1) to maximize the signal‐to‐noise ratio (SNR)^31^, ^32^, ^33^, ^34^, ^35^ at the expense of the maximum achievable depth within the sample, which was 3 mm in this setup. The NMR tube containing the sample was put on the top surface of the device.

**FIGURE 2:**
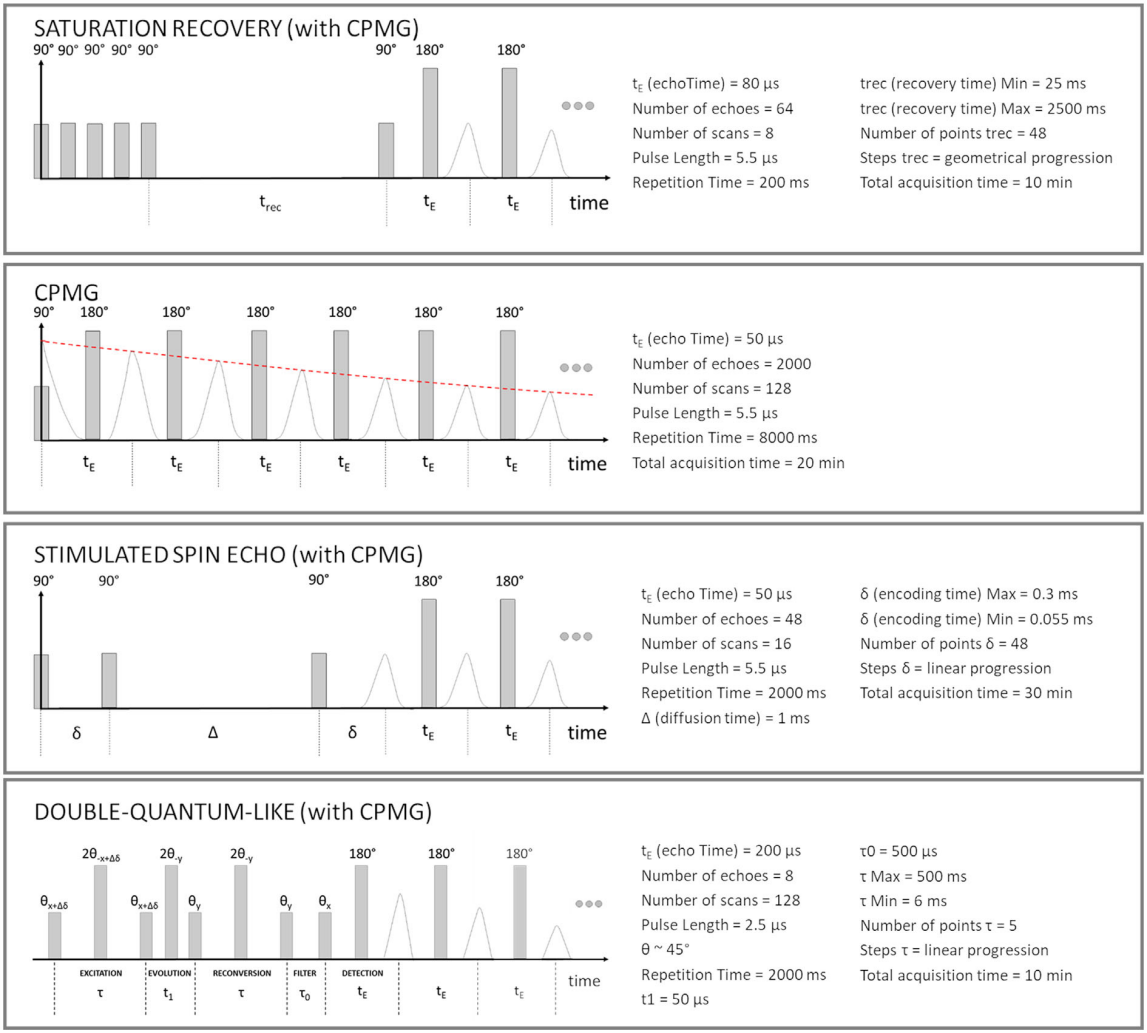
NMR sequences, timing diagrams, and relative parameters. The double‐quantum‐like sequence and the phase cycle used are the same in^36^. An average on the echoes has been computed for all the sequences following a standard acquisition protocol with NMR‐MOUSE. NMR, nuclear magnetic resonance.

The NMR measurement protocol comprised four NMR sequences: Carr–Purcell–Meiboom–Gill (CPMG), saturation recovery (with CPMG), stimulated spin echo (with CPMG), and build‐up DQ‐like (with CPMG)^34^, ^36^, ^48^ sequences to estimate *T*_2_, *T*_1_, *D*, and *α* parameters, respectively (see below for the definition of *α*). The timing diagrams of the sequences and the acquisition parameters of each sequence are reported in Figure 2. All the measurements were performed at room temperature, which was kept stable at 22°C. For all samples selected for the multi‐parametric study, the profile of the signal was acquired depth‐wise inside the cartilage, and the four parameters were measured at the center of the three layers of the cartilage.

### 2.3 Preparation of different setups of the sample inside the NMR tube

The preliminary part of the study was devoted to finding the optimal choice of the experimental setup to minimize changes in the cartilage tissue due to dehydration during the NMR measurements. Different setup configurations were tested, with particular attention to comparing two setups (setup 1 and setup 2). For setup 1, the sample was inserted into a cylindrical glass tube, which was sealed on top with a cap with a wet sponge. The bottom extremity was directly placed over a glass slide resting on the base of the lift. For setup 2, the same steps of setup 1 were used but with the addition of thin layers of polytetrafluoroethylene (PTFE), commercially known as Teflon, to the glass tube extremity that was in direct contact with the cartilage surface. This was meant to further reduce dehydration during the experiment (See Supplementary Material—“Sample Setup Choice” section—Figure S2).

The remaining samples selected for this preliminary part of the study were used to optimize the procedures for measuring the parameters *T*_1_, *T*_2_, *D*, and *α*.

### 2.4 NMR procedure for depth profile acquisition and multi‐parametric zone analyses

**FIGURE 3:**
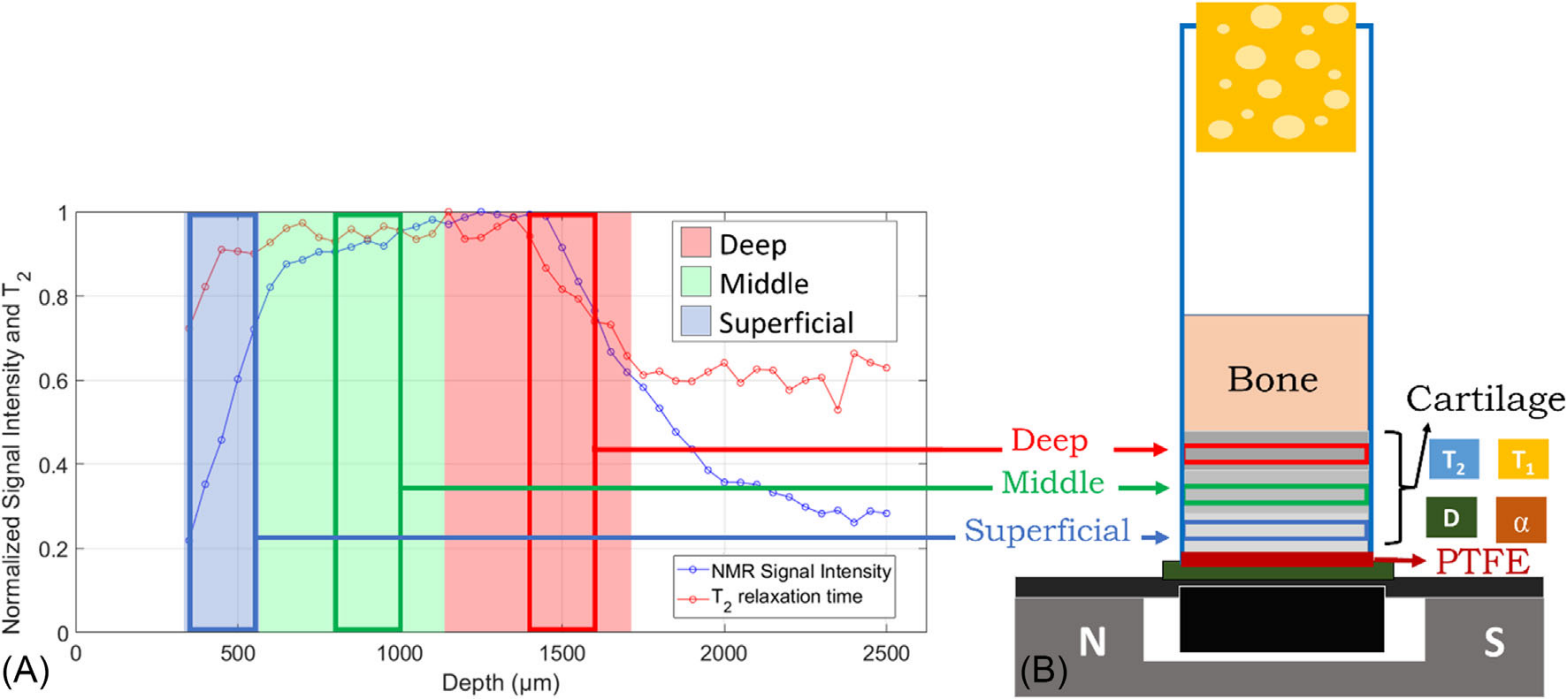
A sketch of the automatized procedure: (left, A) the profile analysis and (right, B) the three‐layer analysis. The first identifies the depths and thicknesses of the superficial (blue), middle (green), and deep (red) cartilage zones of the sample. The second uses the information obtained in the first step to place the sensitive volume of the NMR‐MOUSE PM10 in each cartilage layer, performing the NMR measurements. NMR, nuclear magnetic resonance.

By adopting the sample configuration chosen as described later to perform the multi‐parameter experiment, for each sample, the NMR pulse sequences, described in Section 2.2, were collected in a semi‐automated procedure, organized into two consecutive steps: (i) the profile analysis and (ii) the three‐layer analysis, represented in Figure 3A,B, respectively. The profile analysis allows the determination of the depth and thickness of the three anatomical cartilage zones of the sample: superficial, middle, and deep (user‐dependent step). Starting from the maximum depth allowed by the device configuration, the profile was created by automatically applying a CPMG sequence at different depths within the sample, exploiting the high‐precision lift of the NMR profiler with a 50‐μm step size in‐depth and setting echo time to 91.5 μs corresponding to a slice thickness of 75 μm (acquisition filter on the echo^32^). The CPMG profile was acquired in about half an hour. From CPMG data, both the signal intensity and the *T*_2_ profiles were normalized by their maximum values and represented in the same plot, see Figure 3. In step (i), the fast CPMG acquisition (about half an hour) was characterized by a low SNR, and this determined lower accuracy in estimating the *T*_2_ than the signal intensity. Considering the signal intensity profile, the regions of initial increase, maximum, and subsequent decrease towards the calcified zone have been identified as superficial, middle, and deep layers, respectively (blue, green, and red zones in Figure 3). The three‐layer analysis was then performed. The four NMR sequences, described in Section 2.2, were run by positioning the sensitive volume of the instrument at the center of each layer, with a thickness of 210 μm. It is worth highlighting that the NMR profile provides information on a volume (flat slice) and the collected signal can derive from nonuniform and partially overlapping zones.^51^ The entire procedure for this analysis required 210 min for each sample, and it was made automatic to be more user‐friendly and less user‐dependent.

Many experiments on test samples have been performed to exclude systematic deviations of the four parameters (*T*_1_, *T*_2_, *D*, and *α*) related to any change of the device performances in depth and/or in the temporal execution of the procedure.

### 2.5 Data processing and analysis

Preliminary measurements performed on the three layers of cartilage to set up the experimental conditions (Sections 2.2 and 2.3) showed monomodal quasi‐continuous distributions for the three parameters *T*_1_, *T*_2_, and *D* (see Supporting Figure S3). The quasi‐continuous distributions were obtained by the UPENwin^52^ software based on the UPEN principle.^53^

A monoexponential fit was used on each of the three zones to estimate *T*_1_ and *T*_2_ according to Equations 1 and 2, respectively. A log‐linear fit was used to estimate *D* according to Equation 3, where *γ* is the gyromagnetic ratio and *g* is the field gradient. Refer to Figure 2 for the description of symbols.

(1) $$S\left(t_{\mathrm{rec}}\right)=S_{0}\cdot \left(1-e^{-\frac{t_{\mathrm{rec}}}{T_{1}}}\right)+c_{1}$$

(2) $$S\left(t_{E}\right)=S_{0}\cdot e^{-\frac{t_{E}}{T_{2}}}+c_{2}\;$$

(3) $$\ln\left(\frac{S\left(\delta \right)}{S\left(\delta_{\mathrm{MIN}}\right)}\right)=-D\cdot \gamma^{2}g^{2}\delta^{2}\;\left(∆+\frac{2}{3}\;\delta \right)$$

(4) $$S\left(\tau \right)=\alpha \cdot \tau +c_{3}\;$$

For the DQ‐like sequence, a linear fit was performed on the acquired data according to Equation 4, which allowed the determination of the slope *α*. The fitting procedure was performed using in‐house scripts written in MATLAB (MathWorks, MA, USA). At the end of the data processing procedure, for each sample, four parameters, *T*_2_, *T*_1_, *D*, and *α*, were obtained for each cartilage zones. An example of experimental data and relative fitting is given in Figure S4.

Regarding the DQ‐like buildup sequence, it was chosen because it is potentially useful in bringing out features related to even the macromolecular component of the tissue (i.e., collagen). However, given the impossibility of obtaining quantitative parameters using the timing of excitation and reconversion (*τ*) of the sequence described in the literature,^34^, ^36^, ^48^ *τ* was extended by two orders of magnitude to investigate the possible sensitivity of this sequence to the structural and compositional variations of cartilage tissue. *S*(*τ*) showed a linear dependance on *τ* (Equation 4) which allowed us to define the parameter *α* as the slope of build‐up of the magnetization.

We performed a Kolmogorov–Smirnov test to check whether the distribution of the four parameters among the 20 samples was normal. Then, once the nonnormality was found, to test for statistical difference in each NMR parameter across cartilage zones, the Kruskal–Wallis (KW) test was applied using the data from the 20 samples analyzed. To correct for multiple testing, a post hoc comparison performed using false discovery rate (FDR) was applied instead of a Bonferroni correction because layer populations are not independent of each other. A *p*‐value less than 0.05 was set as the threshold for statistical significance.

Principal component analysis (PCA) was also used as multivariate statistical analysis to test if various combinations of NMR parameters could better separate cartilage layers and explore whether natural clusters would occur in our data. To quantify the clustering effectiveness, a single Silhouette score^54^ was computed as the sum of the three scores related to the 3 clusters of the analysis (superficial, middle, and deep layers clusters).

## 3 RESULTS

### 3.1 Signal intensity and *T*_2_ depth profiles

**FIGURE 4:**
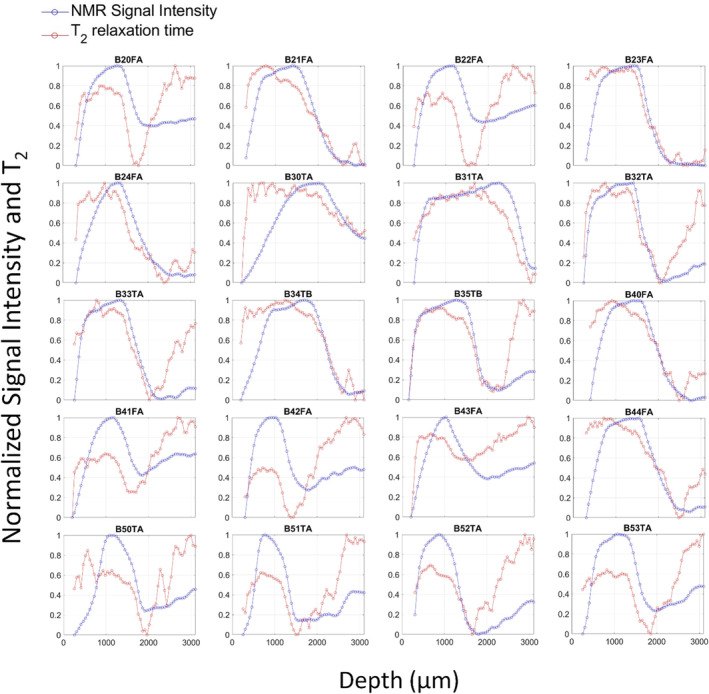
Signal intensity and *T*_2_ profiles acquired on all the 20 samples. Each point is normalized by setting the minimum value to 0 and the maximum to 1 for each plot. *T*_2_ values for NMR profiles ranged within the values reported in Table S1. NMR, nuclear magnetic resonance.

Figure 4 shows both signal intensity and *T*_2_ acquired during the profile analysis. The profiles of the NMR signal for all the 20 samples replicated what was described in ref,^40^ allowing for the identification of the three layers. This gave further control on the ability of the configuration setup 2 to preserve the hydration of the sample. The high variability shown by *T*_2_ profiles depends on the low SNR of fast CPMG sequences used for profiling. The signal intensity showed a lower variability because it was obtained by data extrapolation to 0 time. The increase of *T*_2_ after the region of the deep layer showed by all samples likely originates from mobile protons inside the trabecular and subchondral bone,^40^

### 3.2 Multiparametric depth‐wise NMR analysis

**FIGURE 5:**
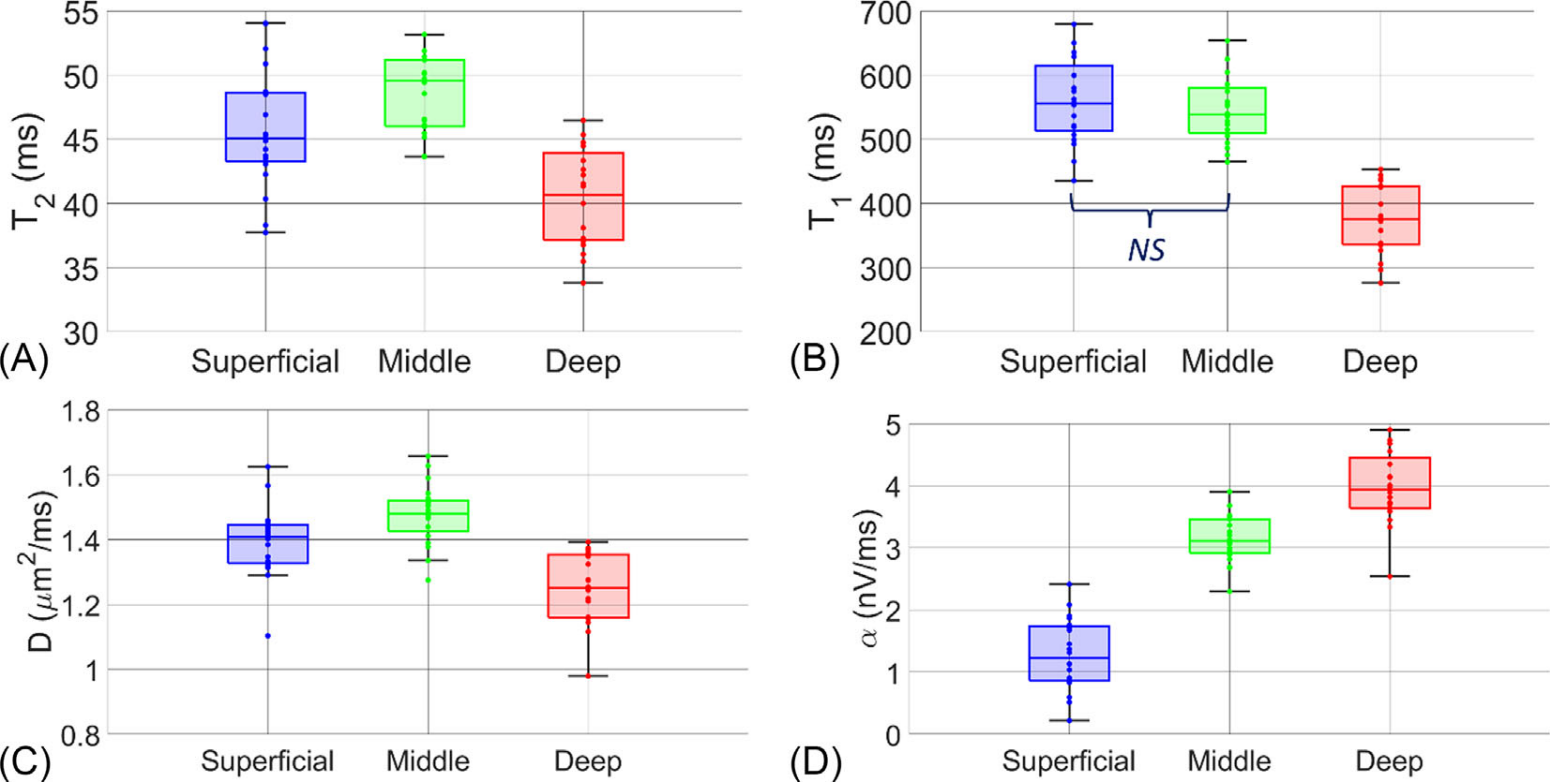
(A) *T*_2_, (B) *T*_1_, (C) *D*, and (D) *α* values boxplots among cartilage layers for all the 20 animal articular cartilage samples. The boxes indicate the 25th and 75th percentiles, and the line within the boxes marks the medians. Whisker length is equal to 1.5× interquartile range. Circles represent outliers. As reported in Table 1, there is no statistically significant difference only in the *T*_1_ values for the comparison between the superficial and middle layer (NS) NS, not significant.

The values of the four parameters *T*_1_, *T*_2_, *D*, and *α*, evaluated at the center of the three cartilage zones obtained on all the 20 samples, are not normally distributed, and they are shown in the box plots of Figure 5 and in Table S1.

Qualitatively, for *T*_2_, *T*_1_, and *D*, the deep layer medians were lower than the respective medians in the superficial and middle layers (see Figure 5A,B,C, respectively). Concerning the α parameter (Figure 5D), the values monotonically increased moving from the superficial to the deep layers. To get some insight about the origin of the behavior of α, we have investigated the dependence of this parameter on the others. It has been proven that the signal increase as a function of τ is strongly related to both the total magnetization (*S*_0_) and the longitudinal relaxation time (*T*_1_) of the system. Considering the whole dataset of this work (with α in nV/ms, *T*_1_ in *s* and *S*_0_ in mV), *α* showed a strong linear correlation with *S*_0_ and *T*_1_ and the following relationship has been found *α* = − 4.4 *T*_1_ + 2.9 *S*_0_ + 2.1 (*R* ^2^ = 0.967).

For this analysis, we used *S*_0_. This parameter was not used in the previous steps of the analysis as an indicator of the water content for a technical reason that could affect *S*_0_ (*S*_0_ could be underestimated in the superficial layer not for a lower ^1^H content but by the imperfect alignment of the sensitive volume with respect to the surface of the sample). Moreover, *S*_0_ is the signal from both water and PG, so, in any case, it could not be a parameter for quantifying the water content.

On the contrary, for the analysis of *α*, we used just *S*_0_, considering that *S*_0_ could significantly change not only with the water concentration but also with the PG concentration inside the sensitive volume. For this reason, due to the possible dependence of *α* on the presence of PG, *S*_0_ could be interesting to understand the origin of this new parameter. The observed largest *α* values (deep zone) corresponded to the maximum values of the signal (*S*_0_) and the minimum values of *T*_1_. Therefore, *α* parameter has a different information content than the other parameters singularly taken because it is also related to the complex mechanisms of magnetization and/or chemical exchange among protons of water, PG, and collagen that influence both *S*_0_ and the longitudinal relaxation.

The results of the analysis of variance performed with the KW test and post hoc comparisons are reported in Table 1. Except for one comparison (*T*_1_ values between superficial and middle layers), for all four parameters, a *p*‐value < 0.05 of statistical significance was found for differences in parameter values among layers.

**TABLE 1:** Results of a statistical analysis based on the KW test.

NMR parameter	Layer comparisons	KW *p*‐values	
*T*_2_	*Superficial‐middle‐deep*	<0.0001	‐
	*Superficial‐middle*	0.0284	‐
	*Superficial‐deep*	0.0009	‐
	*Middle‐deep*	<0.0001	‐
*T*_1_	*Superficial‐middle‐deep*	<0.0001	‐
	*Superficial‐middle*	0.4989	*NS*
	*Superficial‐deep*	<0.0001	‐
	*Middle‐deep*	<0.0001	‐
*D*	*Superficial‐middle‐deep*	<0.0001	‐
	*Superficial‐middle*	0.0045	‐
	*Superficial‐deep*	0.0002	‐
	*Middle‐deep*	<0.0001	‐
*α*	*Superficial‐middle‐deep*	<0.0001	‐
	*Superficial‐middle*	<0.0001	‐
	*Superficial‐deep*	<0.0001	‐
	*Middle‐deep*	<0.0001	‐

^NS^ : not significant difference after correction for multiple comparisons.

**FIGURE 6:**
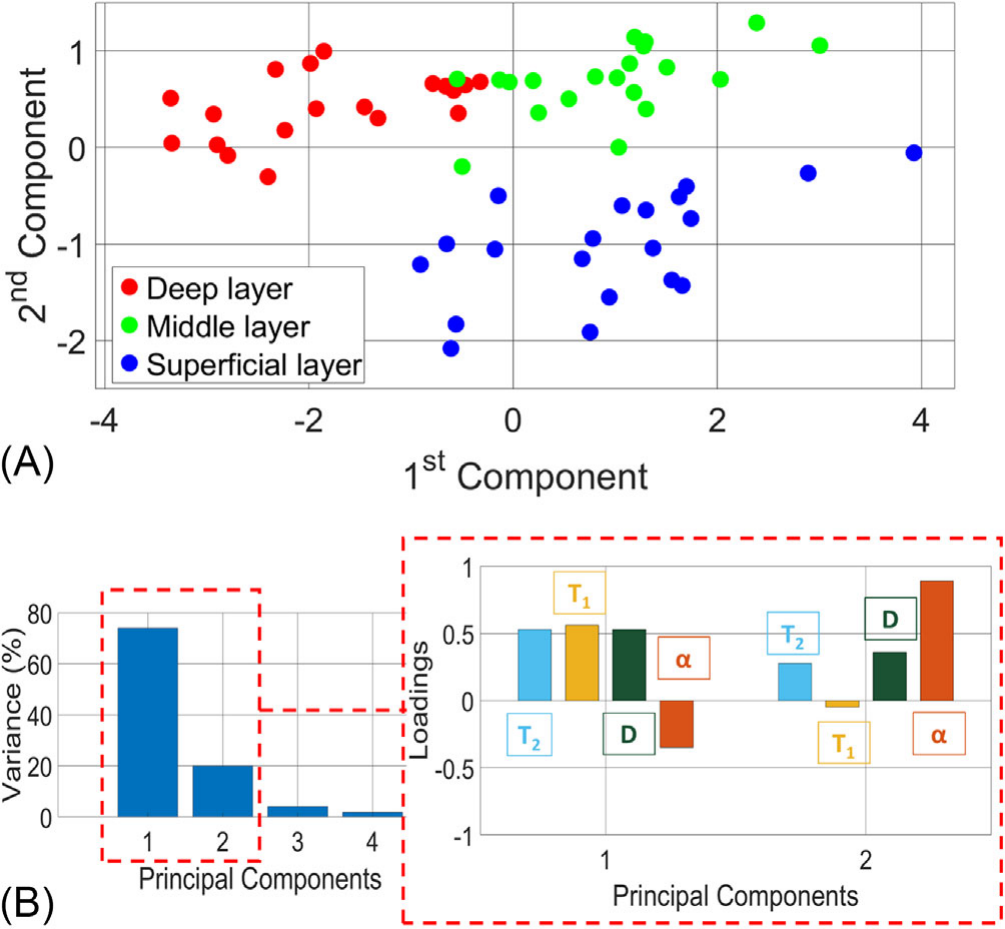
(A) Principal component analysis (PCA) results: the score plot of the first two components is colored according to the three cartilage layers. (B) Percentage of the total variance related to each component and the loadings for the first two selected components.

Figure 6 summarizes the results of the PCA analysis. The scatter plot produced using the first and second principal components from the PCA analysis is reported in Figure 6A. The scatter plot shows that three clusters corresponding to the three cartilage layers originated by combining all four parameters. Considering the four parameters, the clustering performance was scored as 1.33 (Silhouette). By looking at the variance explained by each principal component, reported in Figure 6B, the first two components explained 94% of the total variance: 74% the first and 20% the second. Investigating the correlations between each NMR parameter and the principal component value (box insertion in Figure 6B), it was possible to observe that the first principal component had a positive correlation to *T*_2_, *T*_1_, and *D* variables (*r* values were equal to 0.53, 0.56, and 0.53, respectively), and a negative correlation with *α* (*r* = −0.35). The second component, instead, had the highest positive correlation with the *α* variable (*r* = 0.89) and lower correlations with *T*_2_, *T*_1_, and *D* (*r* values were 0.28, −0.05, and 0.36, respectively).

The PCA was repeated using all possible combinations of parameters and trying to exclude some of them in turn. In some cases (among the comparisons: superficial vs. middle, superficial vs. deep, and middle vs. deep), using two parameters rather than four for the PCA leads to better clustering in terms of Silhouette score. Two of the best results were obtained by considering *T*_1_ and *α* (score = 1.79), and by considering *D* and *α* (score = 1.56).

## 4 DISCUSSION

This study has been performed on excised animal samples. The ex vivo NMR multiparametric preclinical analysis performed by a low‐field single‐sided device presents itself as an especially useful step to better understand the origin and the potentiality of using *T*_1_, *T*_2_, or other NMR parameters in clinical applications. As well known, the interpretation of the origin of NMR parameters in AC is made difficult by the complexity of the tissue structure and composition. In each zone, the distribution, form, and size of the chondrocytes, the content of PG, and the density and orientation of the collagen fibrils differ. Ex vivo analyses are limited as they cannot effectively catch the macroscopic characteristics of the cartilage. However, excision does not affect microscopic structure and fiber orientation, which NMR can successfully study, as discussed in ref.^29^ Thus, ex vivo NMR studies offer important tools in preclinical research to correlate the observed variations of NMR parameters with AC changes in the presence of different diseases and their progression. An important drawback of ex vivo analysis is sample dehydration during NMR measurement. To maximize the meaningfulness of information obtained using ex vivo samples, it is crucial to maintain the original hydration as much as possible throughout the experimental measurement. To this aim, in the present study, great care was devoted to optimizing the specimen excision, storage, and treatments.

The setup adopted in this study was chosen among other procedures to exhibit the best trade‐off between ex‐vivo extraction and analysis of the sample, preservation of sample hydration, and values of NMR parameters. Also, the use of the whole osteochondral unit (envisioning future mechanical analysis of the whole package of articular tissues) could have had the collateral benefit of reducing the dehydration of the sample. It is worth quoting that the depth profile acquired on all the AC samples was found in good agreement with the literature,^41^, ^42^, ^43^, ^45^ with a clear distinction between surface, middle, and deep layers through the increase, stability, and decrease of NMR signal intensities (Figure 5).

The high number of samples considered in our data set allows to estimate the scatter of each parameter, and consequently to evaluate the statistical significance of the differences.

The results showed that the four parameters *T*_1_, *T*_2_, *D*, and *α*, considered individually, presented statistically significant differences across layers. The only exception was *T*_1_, which did not show differences between the superficial and middle layers. No significant differences were found between NMR parameters measured in tibial versus femoral cartilage samples.

The PCA analysis showed that the three parameters, *T*_1_, *T*_2_, and *D*, had high comparable loading factors on the first component. The addition of the parameter *α*, which dominates the second component, made it possible to distinguish the three layers in well‐separated clusters. However, it has been verified in our set of samples that the minimum parameter set that could allow the identification of a layer with maximum clustering effectiveness can be composed of only two parameters: *T*_1_ and *α*. In some cases, this option might be optimal in terms of acquisition time rather than taking all four parameters into account. In general, the multivariate analysis (PCA) improved the capability for discriminating the layers by increasing the separation between the superficial and middle ones, as well as the middle from the deep layers (Figure 6).

The discussion of the observed behavior of the four measured NMR parameters and the comparison with the data in the literature deserves special attention, even if the large body of experiments reported in the literature differ due to the diversity of the experimental conditions adopted (hardware, software, origin of the samples …). Also, the strength and the orientation of the polarizing magnetic field with respect to the cartilage surface differ substantially among experiments. Most of the early NMR studies on AC were performed by MRI on a clinical scanner at high magnetic fields (magnetic fields higher than 1 T).

Particular attention should be deserved to the *T*_2_ anisotropy. In ref.^55^, a *T*_2_ anisotropy within the cartilage was observed in MR images and linked to the tissue structure. Images acquired by changing the angle between the direction of the main magnetic field and the surface of the AC (*θ*) clearly show the variation of the signal intensity in the three histological zones (examples in ref.^46^). In ref. ^19^ and ^21^ with μMRI at 7 T, a strong *T*_2_ anisotropy varying with the cartilage depth was observed: the surface and the deep regions showed strong *T*_2_ dependence on the angle between the surface and the magnetic field direction. On the contrary, the parameter *T*_1_ did not show dependence on orientation or layer. At the deep zone, where the collagen fibrils are perpendicular to the articular surface, the percentage decrease of *T*_2_ was found to follow the function (3cos^2^ *θ* − 1)^2^, while little dependence was observed at the transitional zone, where the collagen fibrils are more randomly oriented.^19^

The observations at the high fields of clinical imaging can differ from those obtained at much lower fields. The fields of single‐sided scanners are characterized by high intrinsic gradients but at the same time by lower internal gradients due to susceptibility differences. At the magnetic field of 0.27 T, with a one‐dimensional depth resolution of 50 μm, on bovine AC samples, the *T*_2_ changed from layer to layer, but the dependence on the angle was observed only in samples prepared with 0.8mM aqueous (Gd‐DTPA) solution, within the transition zone.^40^ When the contrast agent was not used, only small and unsystematic variations were observed.^40^ The weakness of this behavior was explained^39^ by some lateral averaging inside the slice over the locally oriented fiber bundles.

In this regard, an interesting aspect can be noticed in Figure 5: the higher variability of the *T*_2_ parameter in the superficial layer respect to the other layers. As expected,^47^ the anisotropy of *T*_2_ in the cartilage is effective only in the superficial layer due to the geometry of the experimental setup. However, each sample was put on the surface of the single‐sided device randomly, without any consideration of a possible angular inhomogeneity. Retrospectively, the variance induced by the anisotropy of *T*_2_ is covered by the variability induced by measurement uncertainty.

At fields lower than those of the clinical scanners, the contrast of *T*_1_ can be larger than at 1 T because the molecular reorientations at lower Larmor frequencies dominate the relaxation.^45^

Our results for the depth dependence of *T*_2_ are consistent with the data available in literature^39^, ^40^, ^41^ (Figure 5), showing an increase from the surface to the middle regions, followed by a clear decrease in the deep layer. *T*_1_ showed this strong decrease, as well, from the middle to the deep layer (Figure 5).

The systematic variations of *D* and the statistically significant lower value in the deep layer with respect to the middle one (Figure 5) provided an interesting new tool of investigation. A systematic significant increase from the surface to the deep layer has been observed for the new parameter *α*. It is the first time that *α* was used to characterize the zones in AC using single‐sided NMR devices, along with *T*_1_, *T*_2_, and *D*. This new parameter seems to be the most efficient to discriminate among layers.

*T*_2_ and *T*_1_ can depend on the surface‐to‐volume ratio of the environments that water molecules can experience by diffusion before relaxing. A specificity of *T*_1_ is also the possible magnetization transfer between collagen and water protons.^18^ In animal collagen samples, *T*_1_ decreased from lower to higher solid‐to‐liquid ratios.^25^

Regarding parameter *D*, its reduction with respect to the bulk water value can depend on the restriction due to physical barriers or/and the intrinsic viscosity of the medium, in our case, the ECM. Moreover, accounting for the effect of diffusion in the static gradient and considering the average values of *T*_2_ and *D* of the middle zone, the observed *T*_2_s were underestimated^41^ by ~25%.

In the experimental conditions adopted (*τ* 2 orders of magnitude higher than literature in the DQ‐like sequence), a relationship has been observed (“Results” section) between *α* and the two parameters *T*_1_ and the equilibrium magnetization of the liquid ^1^H pool. Therefore, the *α* parameter provides additional information content. The variations of the NMR parameters among layers likely depend on the combination of the aforementioned effects with the change in the water and PG concentration and the network of collagen fibers. A sequence similar to the DQ‐like, able to make the signal dependent on the magnetization (*S*_0_) and *T*_1_ as performed in this study, could be used to determine the alpha coefficient on clinical scanners.

In synthesis, relaxation times *T*_1_ and *T*_2_ and diffusion self‐coefficient *D* are sensitive to the concentration and dynamics of the water molecules and their interactions with different cartilage macromolecules (i.e., PG and collagen amide groups), as well as to the interactions with surfaces provided by chondrocytes and collagen fibers.

**FIGURE 7:**
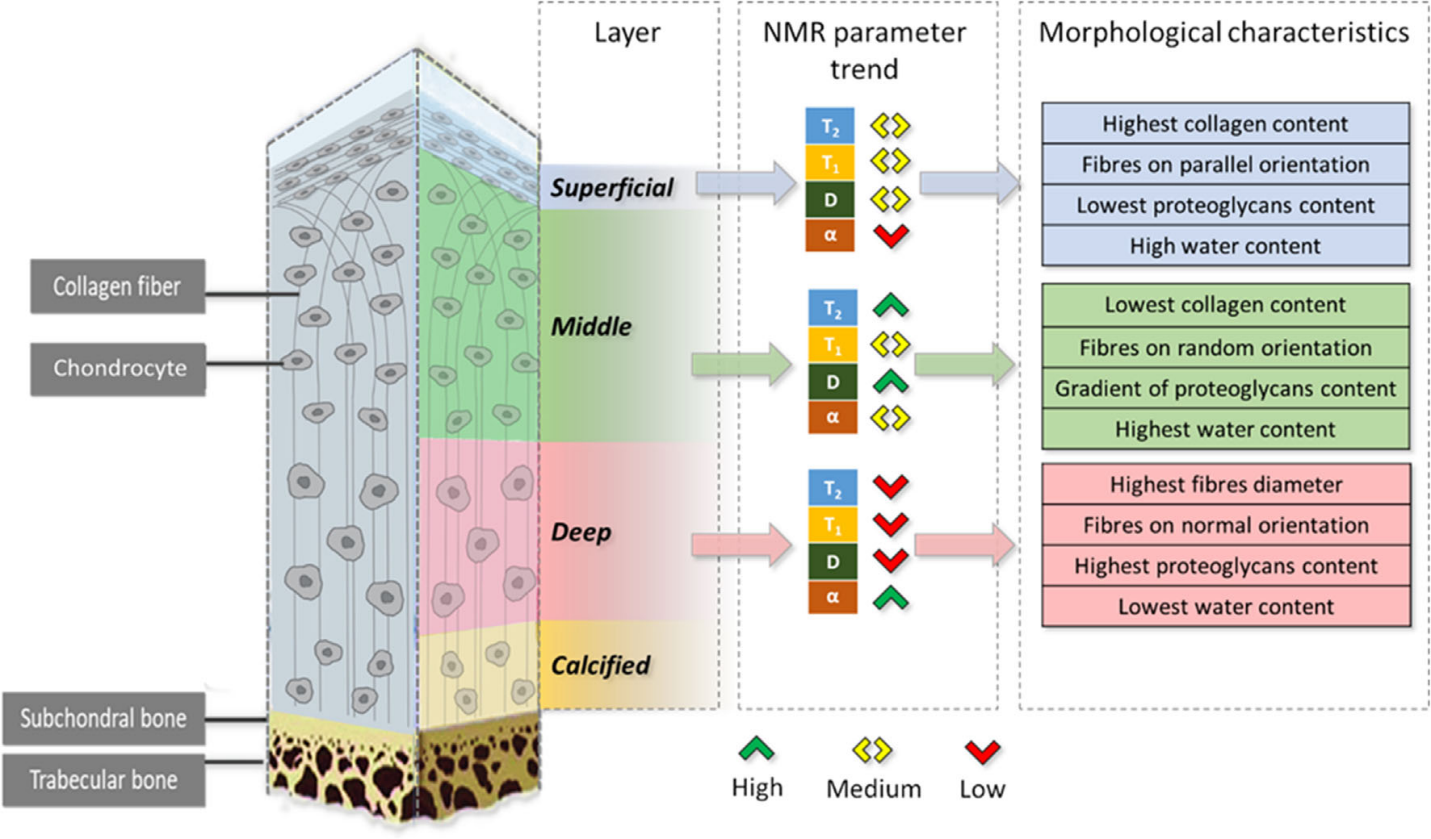
Sketch of the cartilage structure. The measured trends of *T*_2_, *T*_1_, *D*, and *α* values along the layers are reported (^ and v represent the highest and lowest values, while <> is in the middle between these last). The study of cartilage structural changes along zones is possible thanks to the detection of the NMR signal from the ^1^H of water molecules within the tissue. The different organization and content of chondrocytes, collagen, and PG lead to different conditions of water restrictions in the intracellular and extracellular spaces. “Content” means the relative concentration of a cartilage component related to the others considering each zone. Biological information from^1^, ^2^, ^3^. NMR, nuclear magnetic resonance.

In Figure 7, we summarized the trend observed in NMR parameter values across layers, combined with the characteristics of cartilage that might explain such variations.

The changes in these environmental conditions in the three zones can help to understand the trends observed for *T*_1_, *T*_2_, *D*, and *α* moving from one zone to the next. Higher relaxation times and diffusion coefficients occurred in the middle and superficial layers, where the water has the highest concentration and is less restricted by the extracellular matrix compared to the deep layer. In fact, the lowest relaxation times and diffusion coefficient are in the deep layer due to the proximity to the calcified cartilage in which there is the lowest water concentration, and water molecule diffusion is more restricted, likely due to the high PG concentration, that is, the highest in this layer.

The largest values of *α* (deep layer) corresponding to the minimum values of *T*_1_ were observed to be associated to the maximum values of the signal (*S*_0_). This occurs in a zone where the water content is low but there is the highest content of PG (Figure 7). In this complex system, *α* measures the effects of multiple relaxation mechanisms that determine a reduction of *T*_1_ when the signal is high.

The variations of mobility and content of water, content of PG, and collagen structure in the cartilage tissue are suitable indicators for OA severity. Thus, identifying these components through NMR parameters can help in deepening the knowledge about the cartilage changes occurring in this disease. Future studies will also involve unhealthy cartilage and implications with OA, and these results may be integrated with analyses of tissue mechanical properties for a complete evaluation of cartilage changes throughout OA disease.

## 5 CONCLUSION

This work aimed to provide a set of robust NMR parameters able to characterize the laminar structure of AC and investigate the origin of their dependence on the zones. The work was carried out ex vivo on excised animal samples, where the AC was studied without removing it from the osteochondral unit for future mechanical analysis of the whole package of articular tissues. To perform this multi‐parametric depth‐wise characterization, the commercially available low‐field single‐sided NMR device, NMR‐MOUSE PM10, was used, which offered the possibility to scan the depth of the AC with the necessary spatial resolution of 50 μm.

The behavior of the single parameters *T*_1_, *T*_2_, and *D* along the layers of AC is known in literature and confirmed by the present study.

The specificity of this study is manifold. On the one hand, the rather large number of samples analyzed by a multi‐parameter approach allowed to get information not only about the depth dependence but also about the scatter of the four concurrent NMR parameters. The variability of the parameters is of utmost relevance for the study of cartilage tissues and for a possible future goal for the identification of diseases. On the other hand, the large experimental data collection allowed for a statistical analysis able to identify the smallest number of parameters or their combination able to classify samples. A further novelty of this study was the feasibility of applying the build‐up DQ‐like sequence in the study of cartilage with single‐sided NMR.

The systematic variations of the three parameters *T*_1_, *T*_2_, and *D*, with significantly lower values in the surface and the deep layers, when compared with the middle layer, provide an interesting investigation tool. In addition, the novel parameter, *α*, used for the first time in these kinds of studies, appears to be a robust parameter to characterize the layers using single‐sided NMR devices, showing a clear monotonic increase from the surface to the deeper layer. The new NMR parameter *α* appears to be a clear marker of the layered structure. The intuition of using a DQ‐like sequence even if with different timing with respect to the standard approach turned out to be useful but needs further investigation to understand the most important relaxation mechanisms involved.

The results of the present study demonstrate the strong connection between tissue composition and structure of the complex matrix of fibers confining and interacting with water molecules, determining, in particular, a reduction of the self‐diffusion coefficient from the middle to the deep layer.

The discrimination of cartilage layers by multiple NMR parameters was reinforced by performing the multivariate statistical analysis by PCA, maximizing the total variance. The results showed that the use of the first two components was able to cluster the data according to the three different layers. By using two parameters only, the best result was obtained in the present set of data, by considering *T*_1_ and *α*. This result can offer a way to simplify data acquisition and reduce the analysis duration.

On the whole, these results confirmed and highlighted the sensitivity of the NMR parameters to the structural and compositional variations of the cartilage tissue. The discrimination of compositional changes with depth by the four NMR parameters shown in this study and their combination has a very high statistical significance. The single‐sided NMR approach can provide information about NMR parameters connected with tissue composition and structure.

## CONFLICT OF INTEREST STATEMENT

The authors declare no conflicts of interest.

## Supporting information


**Figure S1**: Distributions of T_1_, T_2_, D, and α in the superficial layer. The boxplots represent the data shown in the manuscript (“frozen”), while the markers (red dots) are measures of the “fresh” samples.
**Figure S2:** a) Decrease of the Signal Intensity, normalized to the maximum value for the 2 experimental setups (setup 1 in red, setup 2 in black), obtained through a CPMG sequence. b) Monitoring over time of the four NMR parameters for four samples prepared with the configuration setup 2. Measurement at 700 μm from the cartilage surface.
NOTE: in Figure S2b, T_2_ values could be overestimated due to the fit process (fit evaluated without accounting for the offset parameter and using a linear fit model on the logarithm of the signal).
**Figure S3:** Quasi‐monomodal behaviors for T_1_, T_2_, and D obtained by quasi‐continuous distribution analysis observed on preliminary measurements performed on the three layers of an excised sample of animal articular cartilage.
**Figure S4:** Examples of experimental data and corresponding curves from nonlinear fits of the four NMR sequences. The models used for fitting data are reported in Sect.2.5 of the manuscript. Note: for non‐linear fits, the C.O.D. (Coefficient of determination) that measures the fraction of the total variance of the acquired data accounted for by the model is reported.
**Table S1:** Statistical analysis results for the four NMR parameters among the three cartilage layers.

## Data Availability

The data that support the findings of this study are available from the corresponding author upon reasonable request.
