# Supplementary material for: Depth‐wise multiparametric assessment of articular cartilage layers with single‐sided NMR
Source: NMR Biomed. 2024 Nov 7;38(1):e5287. doi: 10.1002/nbm.5287 (PMC11602265; doi:10.1002/nbm.5287)

*SUPPLEMENTARY MATERIAL*

Depth-wise multi-parametric assessment of articular cartilage layers with single-sided NMR

C. Golini^1^, M. Barbieri^2^, A. Nagmutdinova^3^, V. Bortolotti^3^, C. Testa^1^ L. Brizi^1^

^1^ Department of Physics and Astronomy, University of Bologna, IT

^2^ Department of Radiology, Stanford University, Stanford, CA, United States

^3^ Department of Civil, Chemical, Environmental and Material Engineering, University of Bologna, IT

*Fresh vs. frozen-thawed samples*

Five samples were cored without any freezing-thawed procedure (“fresh” samples). They were measured immediately after the coring procedure. The results were compared to the ones presented in the manuscript (measured after the freezing-thawed procedure - “frozen” samples). For the “fresh” samples, the T_1_, T_2_, D, and α distributions in the three layers were not normal (like in the “frozen” sample distributions), so the Mann-Whitney test was performed to check the differences between “frozen” and “fresh” distributions. The distributions are 12 (4 NMR parameters x 3 cartilage layers): for 9 of them, there is no significant difference between "fresh" and "frozen" samples. The distributions differ in 3 cases only (T_2_ in the superficial layer, D in the superficial and middle layers).

The percentual difference between the median values of “fresh” and “frozen” samples was 6-8% for D in the superficial and middle layers, respectively. The differences were higher for T_2_ in the superficial layer (less than 20%). It is important to note that, on the contrary, T_1_ and α did not show any statistical difference in the superficial layer. As an example, Figure S2 shows the distributions obtained for T_2_, D, T_1_, and α in the superficial layer, showing that the important parameters T_1_ and α of "fresh" and "frozen" samples do not differ in the middle and deep only, but also in the superficial layer. Thus, regarding the freeze-thaw procedure, these results do not invalidate but confirm the results presented and discussed in the manuscript.

*Figure S1: Distributions of T_1_, T_2,_ D, and α in the superficial layer. The boxplots represent the data shown in the manuscript (“frozen”), while the markers (red dots) are measures of the “fresh” samples.*

*
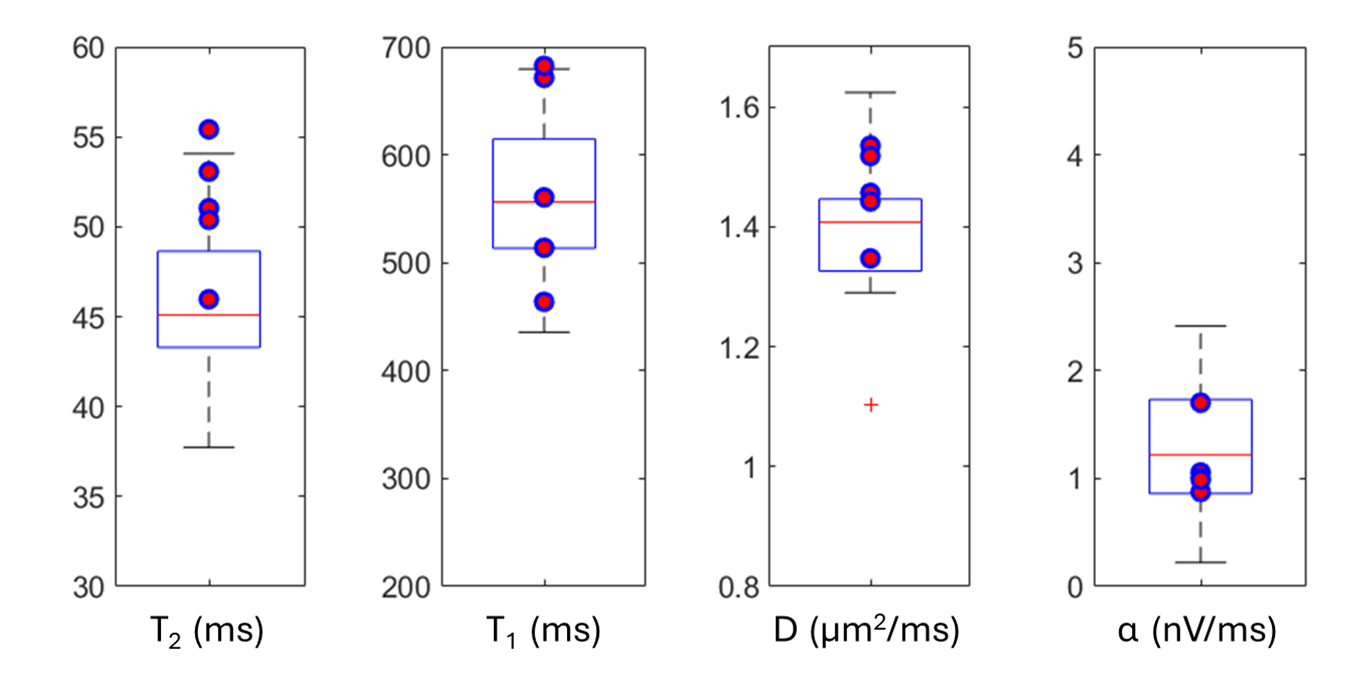
*

*Sample Setup Choice*

The samples prepared by configurations setup 1 and setup 2 were monitored over the time *t_m_* for consecutive CPMG measurements, repeated ten times in 10 hours, to analyze the evolution of both signal intensity *S*_0_(*t*_m_) and *T*_2_(*t*_m_) as indicators of possible dehydration.

An example is reported in Figure S3a, where the changes of *S_0_*(*t*_m_) with respect to the initial value measured at 700 μm from the cartilage surface (middle layer) over time were almost negligible for setup 2 (black dots) while, on the contrary, setup 1 (red squares) showed a clear decrease. Also, in Figure S3b, the substantially constant behavior over time of the four NMR parameters observed in the middle layer confirmed that setup 2 was a good choice to prevent dehydration. Thus, setup 2 was selected as the best choice for performing the following multi-parameter NMR experiment.

Figure S2: a) Decrease of the Signal Intensity, normalized to the maximum value for the 2 experimental setups (setup 1 in red, setup 2 in black), obtained through a CPMG sequence. b) Monitoring over time of the four NMR parameters for four samples prepared with the configuration setup 2. Measurement at 700 µm from the cartilage surface.

NOTE: in Fig. S2b, T_2_ values could be overestimated due to the fit process (fit evaluated without accounting for the offset parameter and using a linear fit model on the logarithm of the signal).

*
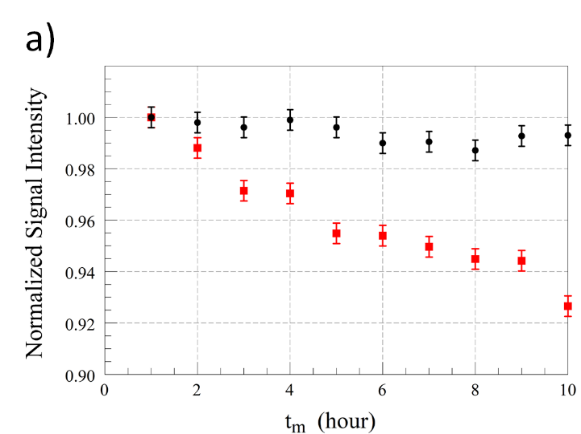
*


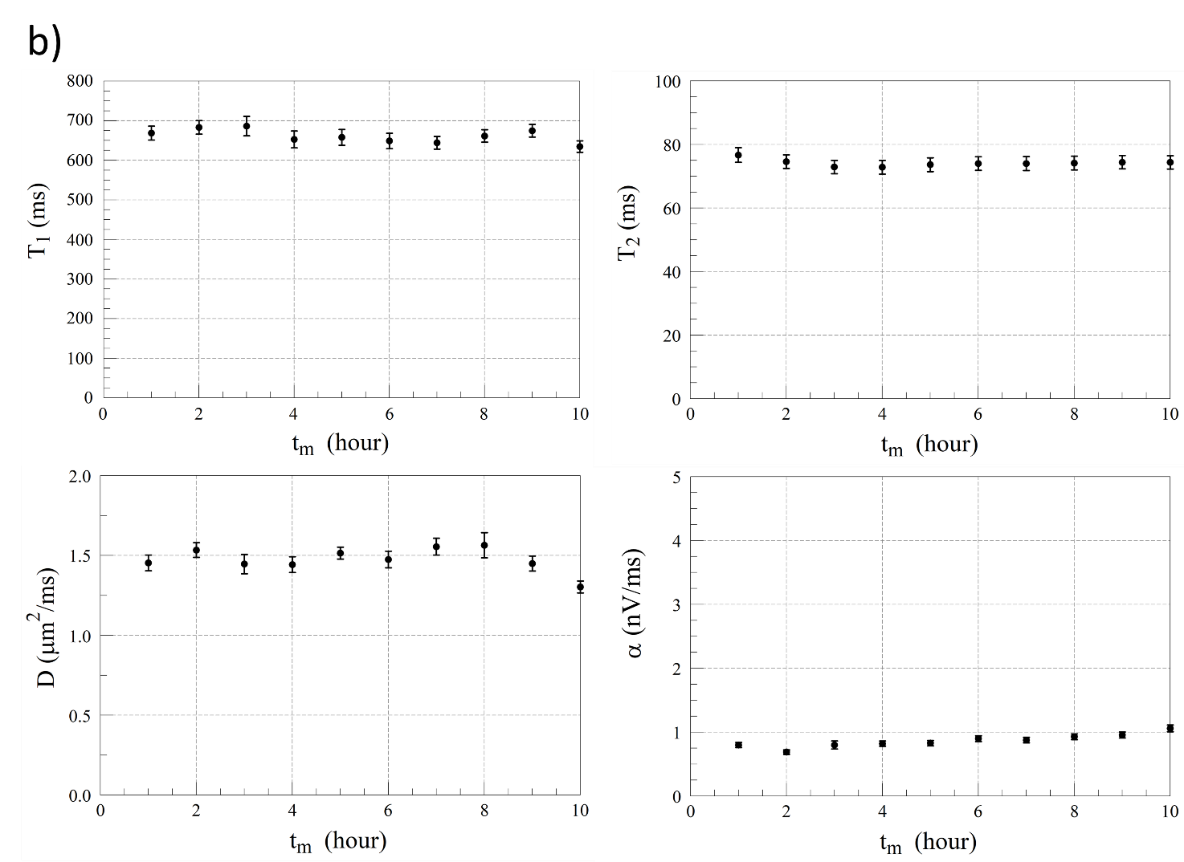


*Figure S3*: Quasi-monomodal behaviors for *T*_1_, *T*_2,_ and *D* obtained by quasi-continuous distribution analysis observed on preliminary measurements performed on the three layers of an excised sample of animal articular cartilage.

*
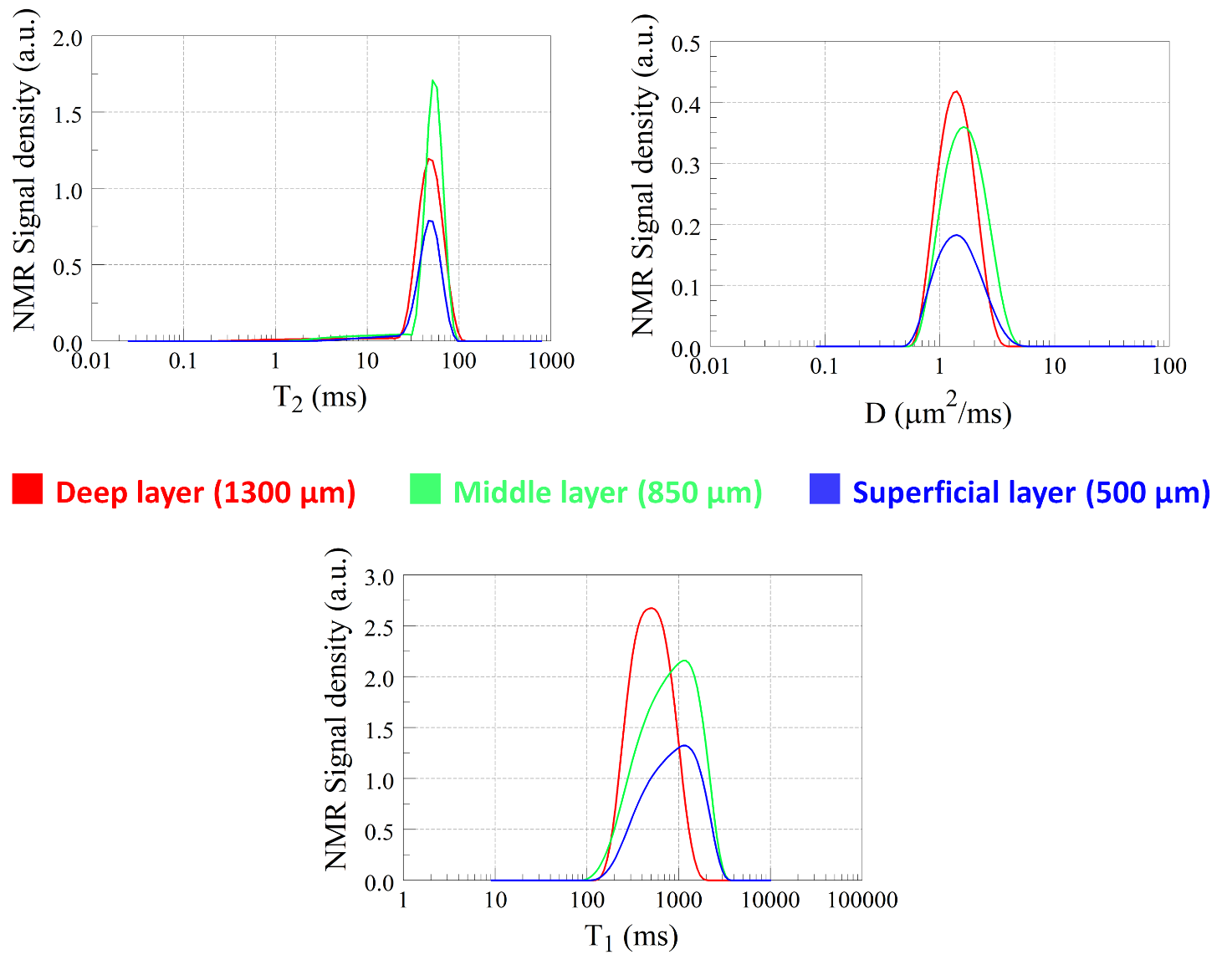
*

*TABLE S1: Statistical analysis results for the four NMR parameters among the three cartilage layers.*

| **Parameter** | **Layer** |  | **percentile** |  |
| --- | --- | --- | --- | --- |
|  |  | **25^th^** | **50 ^th^** | **75 ^th^** |
| T_2_  (ms) | superficial | 43,3 | 45,1 | 48,6 |
|  | middle | 46,0 | 49,6 | 51,2 |
|  | deep | 37,2 | 40,7 | 43,9 |
|  |  |  |  |  |
| T_1_  (ms) | superficial | 513 | 556 | 615 |
|  | middle | 510 | 539 | 580 |
|  | deep | 337 | 376 | 427 |
|  |  |  |  |  |
| D  (μm^2^/ms) | superficial | 1,33 | 1,41 | 1,45 |
|  | middle | 1,43 | 1,48 | 1,52 |
|  | deep | 1,16 | 1,25 | 1,35 |
|  |  |  |  |  |
| α  (nV/ms) | superficial | 0,9 | 1,2 | 1,7 |
|  | middle | 2,9 | 3,1 | 3,5 |
|  | deep | 3,6 | 3,9 | 4,5 |

*Figure S4: Examples of experimental data and corresponding curves from nonlinear fits of the four NMR sequences. The models used for fitting data are reported in Sect. 2.5 of the manuscript. Note: for non-linear fits, the C.O.D. (Coefficient of determination) that measures the fraction of the total variance of the acquired data accounted for by the model is reported.*


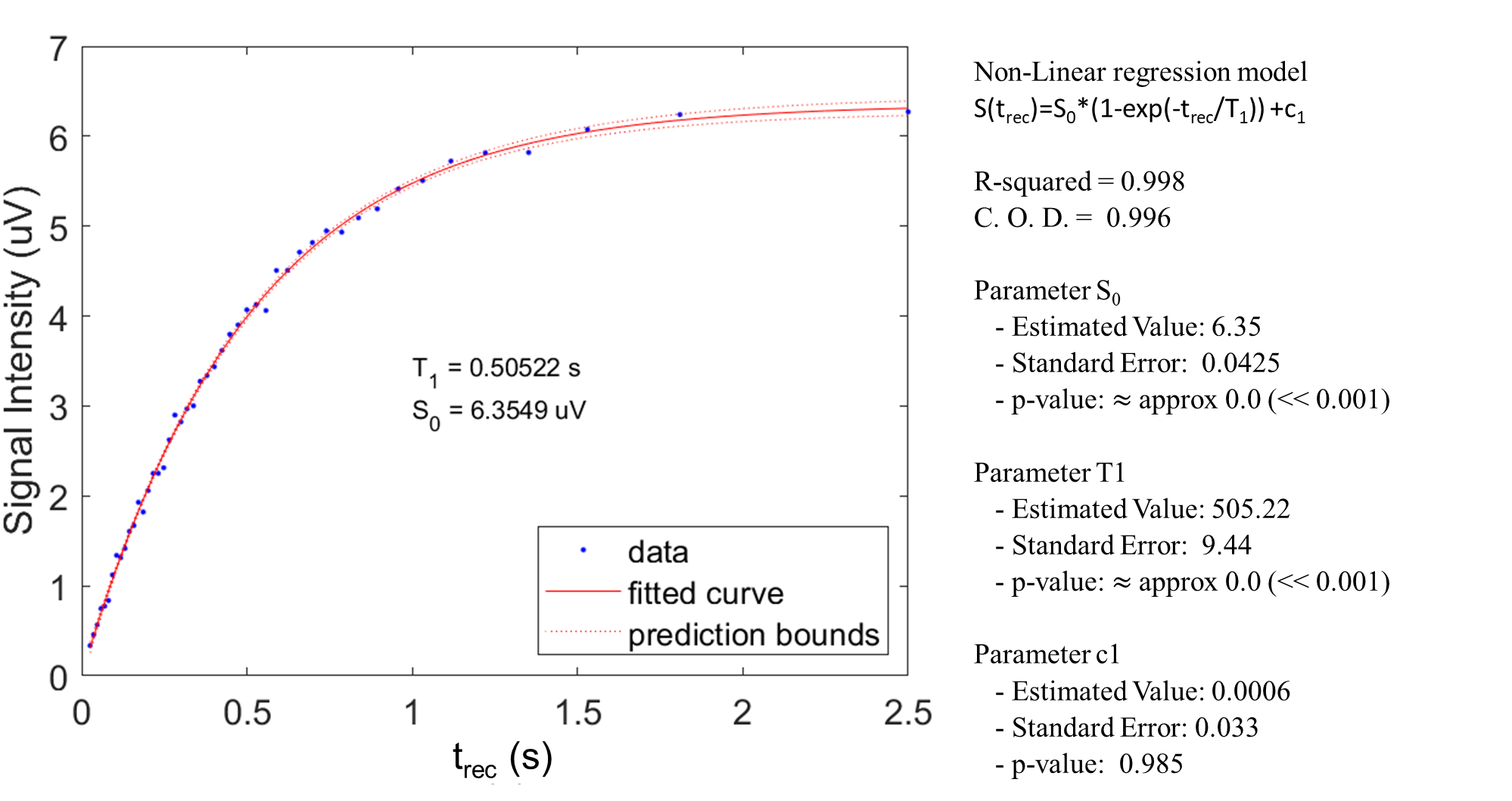


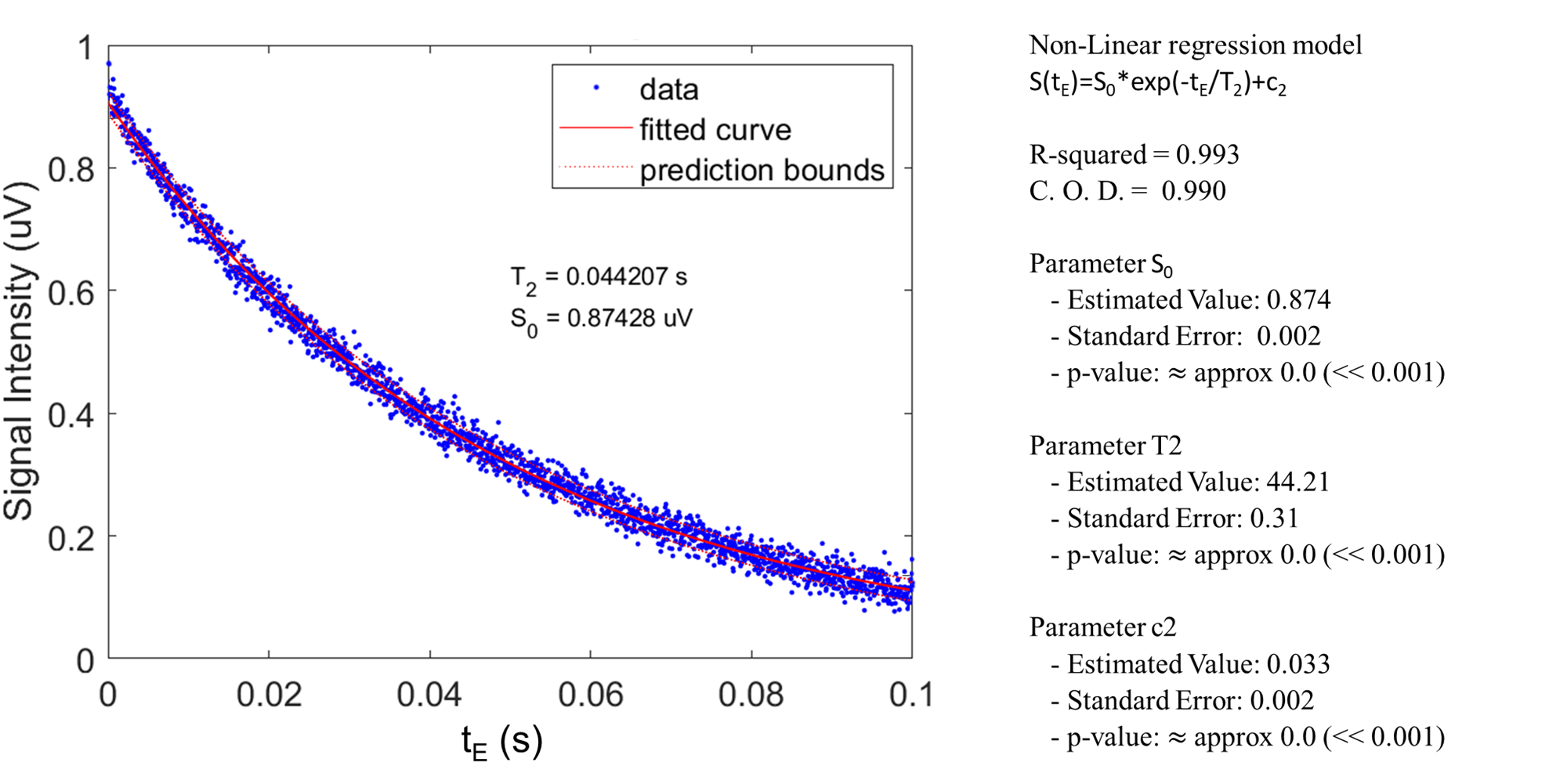


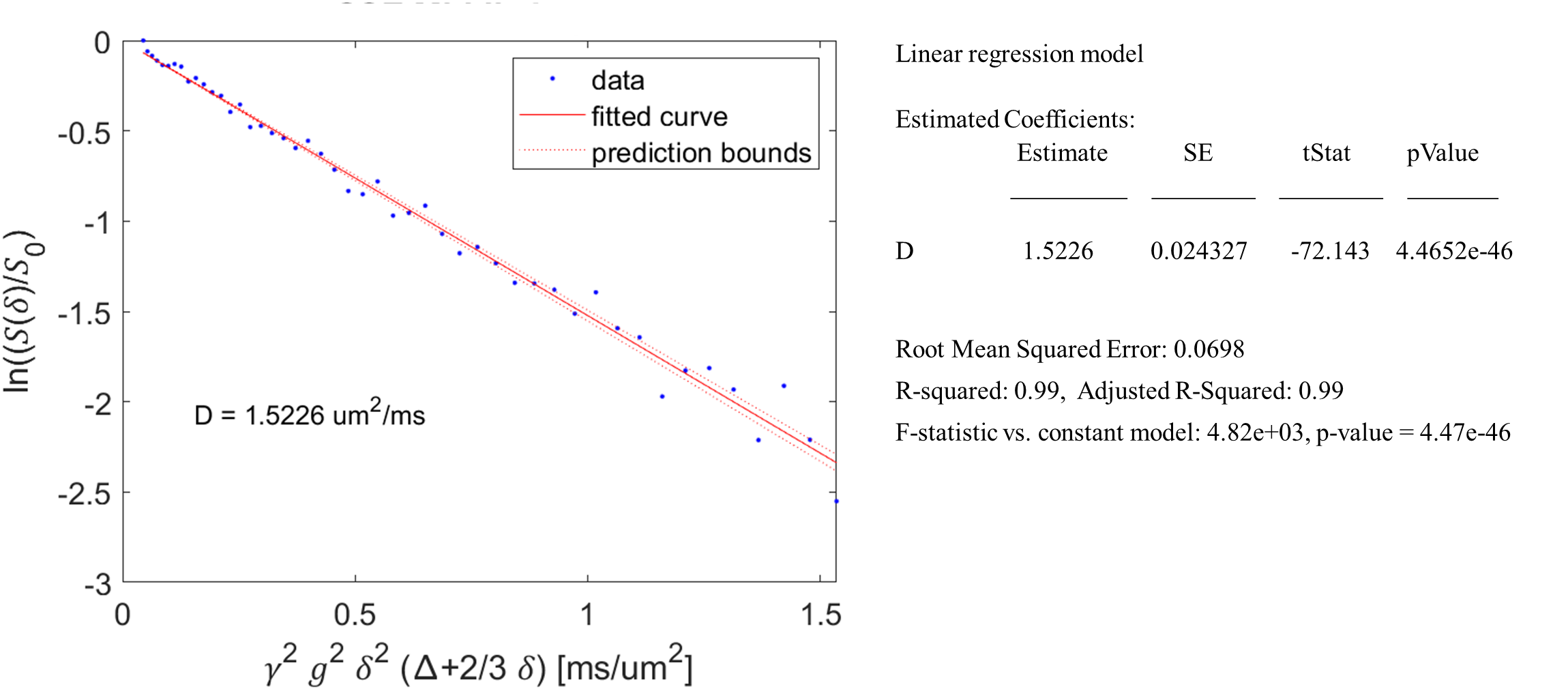


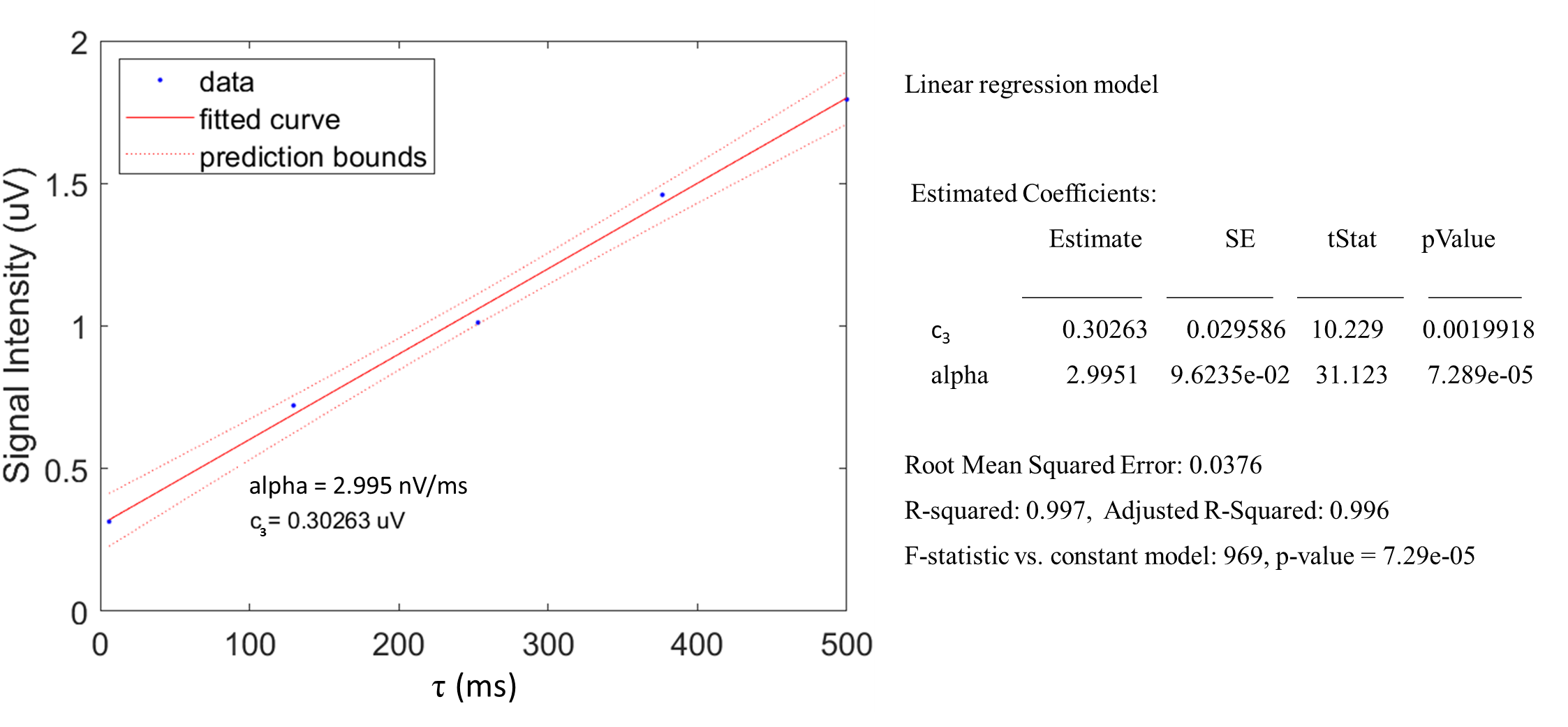

Supplement: Supplementary file 1 — Figure S1: Distributions of T1, T2, D, and α in the superficial layer. The boxplots represent the data shown in the manuscript (“frozen”), while the markers (red dots) are measures of the “fresh” samples. Figure S2: a) Decrease of the Signal Intensity, normalized to the maximum value for the 2 experimental setups (setup 1 in red, setup 2 in black), obtained through a CPMG sequence. b) Monitoring over time of the four NMR parameters for four samples prepared with the configuration setup 2. Measurement at 700 μm from the cartilage surface. NOTE: in Figure S2b, T2 values could be overestimated due to the fit process (fit evaluated without accounting for the offset parameter and using a linear fit model on the logarithm of the signal). Figure S3: Quasi‐monomodal behaviors for T1, T2, and D obtained by quasi‐continuous distribution analysis observed on preliminary measurements performed on the three layers of an excised sample of animal articular cartilage. Figure S4: Examples of experimental data and corresponding curves from nonlinear fits of the four NMR sequences. The models used for fitting data are reported in Sect.2.5 of the manuscript. Note: for non‐linear fits, the C.O.D. (Coefficient of determination) that measures the fraction of the total variance of the acquired data accounted for by the model is reported. Table S1: Statistical analysis results for the four NMR parameters among the three cartilage layers. [file NBM-38-e5287-s001.docx]
